# Sesamin ameliorates nonalcoholic steatohepatitis through inhibiting hepatocyte pyroptosis *in vivo* and *in vitro*

**DOI:** 10.3389/fphar.2024.1347274

**Published:** 2024-02-01

**Authors:** Teng Zhang, Yong Zhou, Yan Zhang, De-Guo Wang, Qiu-Yue Lv, Wen Wang, Ya-Ping Bai, Qiang Hua, Li-Qun Guo

**Affiliations:** ^1^ Department of Gastroenterology, The First Affiliated Hospital of Wannan Medical College, Yijishan Hospital, Wuhu, China; ^2^ Department of Cardiology, Suzhou Hospital of Anhui Medical University, Suzhou, China; ^3^ Department of Gerontology, Geriatric Endocrinology Unit, The First Affiliated Hospital of Wannan Medical College, Yijishan Hospital, Wuhu, China; ^4^ School of Pharmacy, Anhui Innovative Center for Drug Basic Research of Metabolic Diseases, Wannan Medical College, Wuhu, China; ^5^ Anhui Provincial Key Laboratory of Molecular Enzymology and Mechanism of Major Diseases, College of Life Sciences, Anhui Normal University, Wuhu, China

**Keywords:** sesamin, nonalcoholic steatohepatitis, hepatocyte pyroptosis, PKCδ, NLRC4

## Abstract

Sesamin (Ses) is a natural lignan abundantly present in sesame and sesame oil. Pyroptosis, a newly identified type of pro-inflammatory programmed necrosis, contributes to the development of non-alcoholic steatohepatitis (NASH) when hepatocyte pyroptosis is excessive. In this study, Ses treatment demonstrated an improvement in hepatic damage in mice with high-fat, high-cholesterol diet-induced NASH and palmitate (PA)-treated mouse primary hepatocytes. Notably, we discovered, for the first time, that Ses could alleviate hepatocyte pyroptosis both *in vivo* and *in vitro*. Furthermore, treatment with phorbol myristate acetate, a protein kinase Cδ (PKCδ) agonist, increased PKCδ phosphorylation and attenuated the protective effects of Ses against pyroptosis in PA-treated mouse primary hepatocytes. Mechanistically, Ses treatment alleviated hepatocyte pyroptosis in NASH, which was associated with the regulation of the PKCδ/nod-like receptor family CARD domain-containing protein 4/caspase-1 axis. This study introduces a novel concept and target, suggesting the potential use of functional factors in food to alleviate liver damage caused by NASH.

## Introduction

Non-alcoholic steatohepatitis (NASH), characterised by a gradual progression from non-alcoholic fatty liver disease (NAFLD), is a prominent contributor to end-stage liver disease development (Younossi et al., 2019). Our understanding of the pathological mechanisms underlying NASH is currently limited, with treatment options primarily focusing on lifestyle interventions and lipid-lowering therapies (Powell et al., 2021; Paternostro and Trauner, 2022). The absence of effective interventions may lead to the gradual destruction of hepatocytes, resulting in liver fibrosis, cirrhosis and cancer in individuals with NASH (Pouwels et al., 2022).

Pyroptosis, a recently discovered mode of programmed cell death, differs from cell apoptosis (Liu et al., 2016). It relies on the activation of caspase-1 through inflammasomes, leading to the cleavage of gasdermin D into its N-terminal domain (GSDMD-N) and subsequent membrane pore formation (Liu et al., 2016). Notably, pyroptosis has been observed in hepatocytes, and its crucial role in the development and progression of NASH has been demonstrated (Wree et al., 2014; Qiu et al., 2018; Meng et al., 2022).

Sesamin (Ses), a natural lignan found in sesame and sesame oil, possesses various pharmacological properties, including lipid-lowering, liver-protective, antihypertensive and antitumor effects (Li et al., 2013; Majdalawieh et al., 2020; Wen et al., 2022; Yang et al., 2023). Additionally, Ses has been shown to reduce the production of the pro-inflammatory cytokine interleukin (IL)-1β both *in vivo* and *in vitro* (Phitak et al., 2012; Chiang et al., 2014; Fanhchaksai et al., 2016). Inflammasomes promote the activation of caspase-1, leading to the maturation and secretion of IL-1β and, eventually, to pyroptosis. Therefore, we hypothesise that Ses inhibits IL-1β production through the repression of inflammasome activation. The present study aims to investigate whether Ses improves NASH by inhibiting hepatocyte inflammasome activation and pyroptosis both *in vivo* and *in vitro*.

## Materials and methods

### Drugs and reagents

Sesamin (purity: 98%, cat. no.: S25758) was purchased from Yuanye biotechnology (Shanghai, China) (Zhao et al., 2019). Palmitate (PA) was purchased from Kunchuang Co., Ltd. (cat. no.: KC002, Xian, China) (Sun et al., 2023). Phorbol myristate acetate (PMA) was purchased from MedChemExpress (cat. no.: HY-18739, Shanghai, China). The alanine aminotransferase (ALT, cat. no.: C009-2-1), aspartate aminotransferase (AST, cat. no.: C010-2-1), free fatty acids (FFA, cat. no.: A042-2-1), total cholesterol (TC, cat. no.: A111-1-1) and triglyceride (TG, cat. no.: A110-1-1) assay kits were purchased from Jiancheng Bioengineering Institute (Nanjing, China). The lactate dehydrogenase (LDH) release assay kit (cat. no.: C0016), caspase-1 activity assay kit (cat. no.: C1101), calcein-acetoxymethyl ester (AM)/propidium iodide (PI) cell viability/cytotoxicity assay kit (cat. no.:C2015S) and an anti-nod-like receptor (NLR) family CARD domain-containing protein 4 (NLRC4) antibody (cat. no.: AF7578) were purchased from Beyotime (Shanghai, China). The anti-GSDMD (cat. no.: sc-393656), caspase-1 (cat. no.: sc-392736) and IL-1β (cat. no.: sc-52012) antibodies were purchased from Santa Cruz (Texas, United States). The anti-protein kinase Cδ (PKCδ, cat. no.: 9616) and phospho-PKCδ (Thr505, P-PKCδ, cat. no.: 9374) antibodies were purchased from Cell Signaling Technology (Massachusetts, United States). The mouse IL-1β ELISA assay kit (cat. no.: RK00006) and anti-cytokeratin 18 antibody (CK 18, cat. no.: A1022) were purchased from Abclonal (Wuhan, China). The anti-F4/80 antibody (cat. no.: GB11027-100) was purchased from Servicebio (Wuhan, China). The FAM-FLICA^®^ caspase-1 (YVAD) assay kit (cat. no.: 97) was purchased from ImmunoChemistry Technologies (Minnesota, United States).

### Animal experimental protocols

The experimental protocols were thoroughly reviewed and approved by the Animal Ethics Committee of Wannan Medical College. Seven-week-old male C57BL/6 mice, procured from GemPharmatech Co. (Nanjing, China), were allowed a 1-week acclimation period. After acclimatisation, twenty mice (control group) were randomly fed a normal diet (ND), whereas another twenty mice were fed a high-fat, high-cholesterol diet comprising 60% of calories from fat and 2% cholesterol (HFHCD, cat. no.: D190429; Dyets Inc., Wuxi, China). The mice were continuously fed with ND or HFHCD for a total of 14 weeks.

Following 8 weeks of ND or HFHCD feeding, the mice were divided into four groups, each comprising 10 mice: Group I, ND-fed control mice (Con); Group II, ND-fed control mice treated with Ses (Con + Ses); Group III, HFHCD-fed mice (NASH); Group Ⅳ, HFHCD-fed mice treated with Ses (NASH + Ses).

Ses was suspended in 0.5% carboxymethyl cellulose sodium and administered to mice through intragastric gavage for 6 weeks. The daily Ses dosage (120 mg kg^−1^) was determined based on previous studies, ensuring a balance between efficacy and minimal toxicity (Liu et al., 2013; Kong et al., 2015). Mice not receiving Ses treatment were administered an equal volume of carboxymethyl cellulose sodium solution.

Weekly measurements of body weight and food consumption were recorded. At the end of the study, the mice were fasted overnight and anaesthetised through an intraperitoneal injection of sodium pentobarbital, after which their liver weight was measured. The ratio of liver weight to body weight was then determined. Serum and liver samples were collected for biochemical analysis, haematoxylin and eosin (H&E) and Masson’s trichrome staining, immunohistochemical analysis, immunoblotting analysis and IL-1β content detection.

### Intraperitoneal glucose tolerance test (IGTT) and insulin tolerance test (ITT)

The IPGTT and ITT were conducted 1 day before the completion of the study. In the IGTT assay, mice received an intraperitoneal injection of glucose at a dosage of 1.5 g/kg. For the ITT assay, mice were intraperitoneally injected with insulin at a dosage of 0.75 U/kg. Glucose levels were measured using a glucometer (Roche, Shanghai, China) (Xing et al., 2023).

### Biochemical indicator assays and histological and immunohistochemical staining

Liver tissue and serum levels of ALT, AST, FFA, TC and TG were assessed using enzymatic colourimetric reactions with commercially available diagnostic kits. A portion of liver tissues was acquired and immersed in 10% neutral-buffered formalin for 24 h for fixation. Following fixation, the samples were embedded in paraffin and sliced into 4-μm-thick sections. These sections were then subjected to staining, including H&E and Masson’s trichrome, enabling the visualisation of cellular components and tissue morphology. For immunohistochemistry, 4-μm-thick liver tissue sections were incubated overnight at 4°C with an anti-F4/80 antibody, followed by incubation with a secondary HRP goat anti-rabbit IgG antibody. The sections were observed and photographed under a microscope (Xing et al., 2023). H&E-stained liver sections were evaluated using the NASH scoring system (Kleiner et al., 2005). The severity of NASH, determined as the sum of steatosis (graded 0–3) and lobular inflammation (graded 0–3), was assessed by two pathologists using a blinded method. Additionally, the hepatic fibrosis area and the number of F4/80-positive cells were calculated using ImageJ software. At least five random fields at a magnification of ×200 were averaged for each mouse in the liver sections.

### Isolation, culture and treatment of mouse primary hepatocytes

Mouse primary hepatocytes were isolated from eight-week-old male C57BL/6 mice using a two-step perfusion method, as previously described (Gaul et al., 2021; Koh et al., 2021). The primary hepatocytes were cultured in Dulbecco’s modified Eagle medium (Gibco, cat. no.: C11995500BT, New York, United States) supplemented with 10% fetal bovine serum (Gibco, cat. no.: 10099141C), 100 U/ml penicillin, and 0.1 mg/mL streptomycin (Cat. No.: C0222; Beyotime, Shanghai, China). CK 18 immunofluorescence staining was employed to identify primary hepatocytes (Bakhautdin et al., 2014; Debnath et al., 2020).

Primary hepatocytes were seeded at a density of 2.0 × 10^4^ cells/well in 96-well plates (for calcein-AM/PI staining and LDH release assay) or 1.0 × 10^6^ cells/well in 6-well plates (for immunoblotting analysis, IL-1β secretion and caspase-1 activity assays). The primary hepatocytes were stimulated with 0.5 mM PA and either 100 or 50 μM Ses for 48 h (Kong et al., 2015). Additionally, a subset of primary hepatocytes treated with 0.5 mM PA and 100 μM Ses were further stimulated with PMA (200 nM) for 12 h (Tiwari et al., 2011).

### Immunoblotting analysis

In order to extract proteins from mouse liver tissue and primary hepatocytes, we utilized the radioimmunoprecipitation assay lysis buffer. The proteins were then separated through sodium dodecyl sulfate–polyacrylamide gel electrophoresis and promptly transferred onto nitrocellulose membranes. Following this, the membranes were subjected to an overnight incubation with primary antibodies, including P-PKCδ, PKCδ, NLRC4, GSDMD, caspase-1 and IL-1β. The proteins bound by the antibody were visualized using an enhanced chemiluminescence kit**.** Data were analyzed using ImageJ software.

### Caspase-1 activity assay

The activity of caspase-1 in mouse liver tissue and primary hepatocytes was assessed using two different assays. The FAM-FLICA^®^ caspase-1 assay kit, with staining performed using the FAM-YVAD-FMK probe, was applied to frozen liver tissue slides and primary hepatocytes (Pampuscenko et al., 2023). The number of hepatic cells with active caspase-1 was calculated using ImageJ software. For each mouse, frozen liver sections were averaged over at least five random fields at ×200 magnification. The fluorescence of activated caspase-1 staining was measured using a Zeiss LSM 780 confocal microscope (Oberkochen, Germany). Additionally, a colorimetric-based caspase-1 activity assay kit was used to measure caspase-1 activity in primary hepatocytes (Kong et al., 2017). Both assays were conducted following the manufacturer’s protocol.

### LDH release and IL-1β level assays

The supernatants from the mouse primary hepatocytes were transferred and centrifuged at 1,200 rpm for 10 min at 4°C to remove debris. The level of LDH released in the supernatants was measured using the LDH release assay kit, following the manufacturer’s instructions. Additionally, the IL-1β levels in mouse liver tissues and supernatants of primary hepatocytes were determined using an ELISA kit.

### Calcein-AM/PI staining

Mouse primary hepatocytes were co-stained with 100 μL of a testing solution containing calcein-AM/PI, after which the cells were incubated at 37°C for 30 min. The fluorescence signals of calcein-AM, indicating the presence of living cells, and PI, representing dead cells, were assessed using a Nikon Ti fluorescence microscope (Shanghai, China).

### Statistical analysis

Data are expressed as mean ± standard deviation. Differences among multiple groups were analysed using one-way analysis of variance (ANOVA), followed by Tukey’s multiple comparison test. Differences between two groups were assessed using a two-tailed paired Student’s t-test. Statistical significance was defined at *p* < 0.05.

## Results

### Effects of Ses on physical indexes, biochemical indicators, glucose homeostasis, and insulin resistance in NASH mice

**FIGURE 1:**
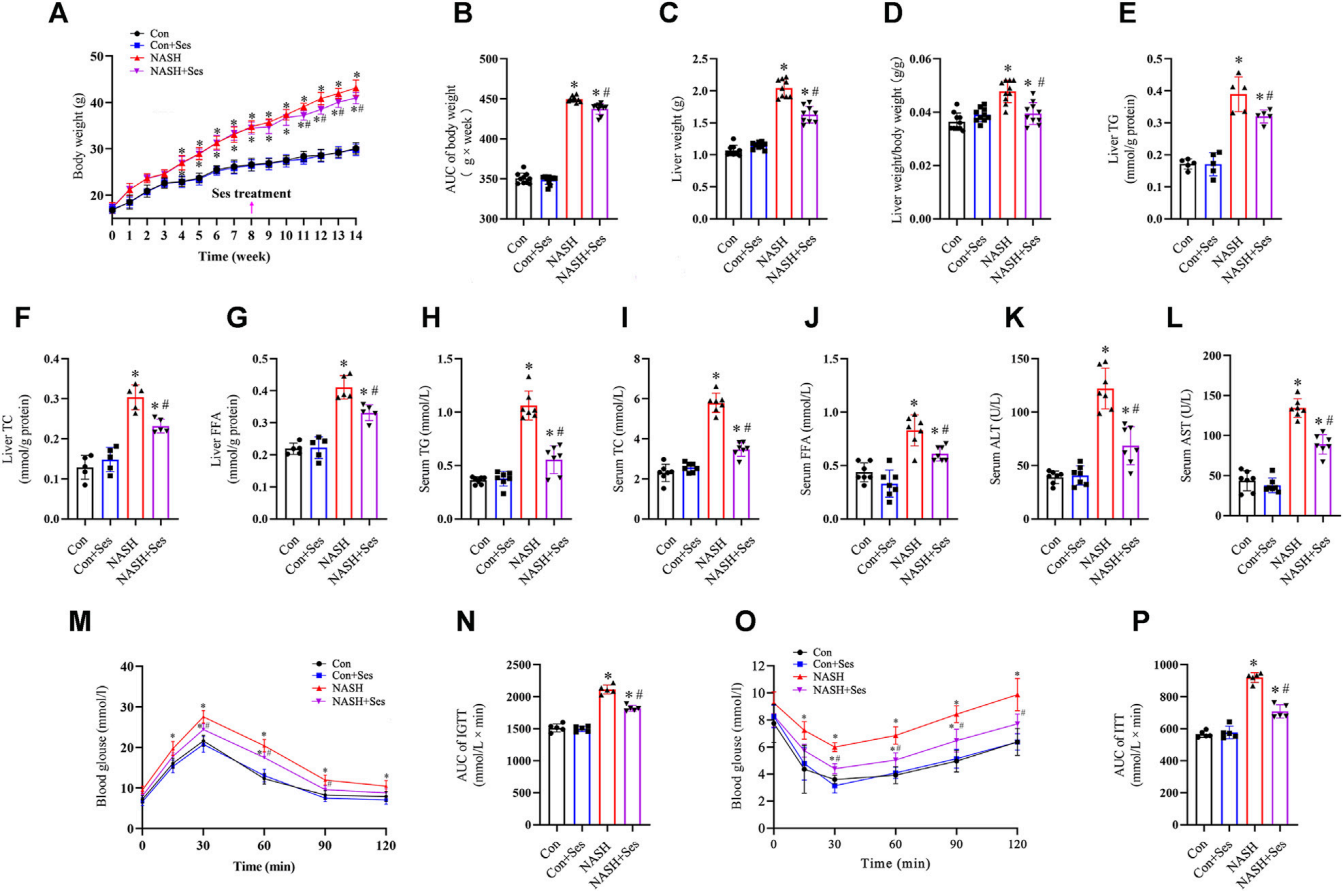
Effects of Ses on physical indexes, biochemical indicators, glucose homeostasis and insulin resistance in NASH mice. **(A)** Body weight was measured weekly (*n* = 10 per group). **(B)** The area under the curve (AUC) of body weight was calculated. **(C)** Liver weight and **(D)** the ratio of liver weight to body weight were measured (*n* = 10 per group). Liver tissue TG **(E)**, TC **(F)** and FFA **(G)** levels were measured (*n* = 7 per group). Serum TG **(H)**, TC **(I)** and FFA **(J)** levels were measured (*n* = 7 per group). Serum ALT **(K)** and AST **(L)** levels were measured (*n* = 7 per group). **(M)** An IGTT was conducted by administering a 1.5 g kg^−1^ glucose injection intraperitoneally (*n* = 5 per group). **(N)** The AUC following the IGTT was calculated. **(O)** The ITT was performed by administering a 0.75 U kg^−1^ insulin injection intraperitoneally (*n* = 5 per group). **(P)** The AUC following the ITT was calculated. *p < 0.05 vs. Con group, ^#^ *p* < 0.05 vs. NASH group.

After 6 weeks of Ses treatment, the NASH mice exhibited a significant reduction in body weight (Figures 1A, B), liver weight (Figure 1C), and the ratio of liver weight to body weight (Figure 1D). Additionally, Ses-treated NASH mice showed reduced levels of FFA, TC and TG in both serum and liver tissue (Figures 1E–J). Administration of Ses for 6 weeks resulted in a notable decrease in the serum levels of ALT and AST in NASH mice (Figures 1K, L). To assess the impact of Ses on glucose homeostasis, an IGTT was conducted. Following glucose loading, Ses treatment significantly reduced the elevated glucose levels in NASH mice (Figures 1M, N). Furthermore, an ITT was performed to evaluate the impact of Ses on whole-body insulin tolerance, showing that Ses treatment significantly improved HFHCD feeding-induced insulin resistance in NASH mice (Figures 1O, P). There were no significant differences in food consumption throughout the experimental period following Ses treatment (data not shown).

### Effects of Ses on hepatic pathological structure and F4/80 staining

**FIGURE 2:**
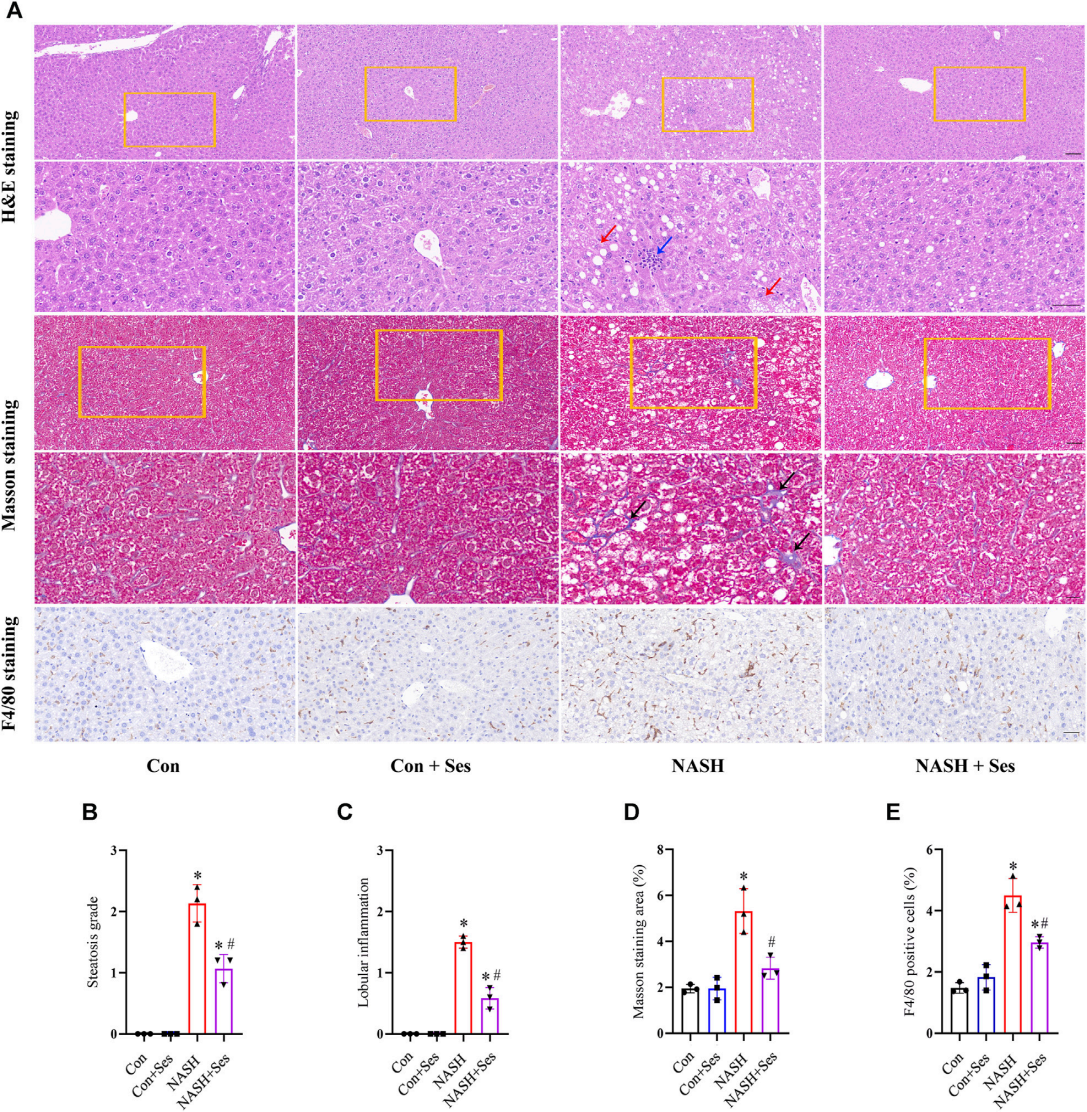
Effects of Ses on hepatic pathological structure and F4/80 staining. **(A)** The liver sections of mice obtained from each group were stained with H&E, Masson’s trichrome and F4/80 antibody. Scale bar from top to bottom: 100, 50, 50, 20, and 50 μm. **(B)** Histologic steatosis and **(C)** inflammation were used to evaluate NASH severity. The red arrow indicates steatosis. The blue arrow indicates inflammatory foci. **(D)** The area of hepatic fibrosis (black arrow) was calculated. **(F)** The number of F4/80 positive macrophages was calculated (*n* = 3 per group). *p < 0.05 vs. Con group, ^#^ *p* < 0.05 vs. NASH group.

H&E and Masson’s trichrome staining were utilised to assess the pathological structure of the mice’ liver (Figure 2A). NASH mice displayed signs of liver damage, including steatosis and lobular inflammation. Ses administration significantly alleviated these pathological indications of liver injury in NASH mice (Figures 2B, C). Hepatic fibrosis was observed in NASH mice, as depicted in Figure 2D, and Ses treatment led to its improvement. Additionally, F4/80 staining was employed to indicate the number of macrophages in the mice’ liver. Ses treatment significantly reduced the number of hepatic macrophages in NASH mice (Figure 2E).

### Effects of Ses on hepatic PKCδ-induced NLRC4 inflammasome activation and pyroptosis in NASH mice

**FIGURE 3:**
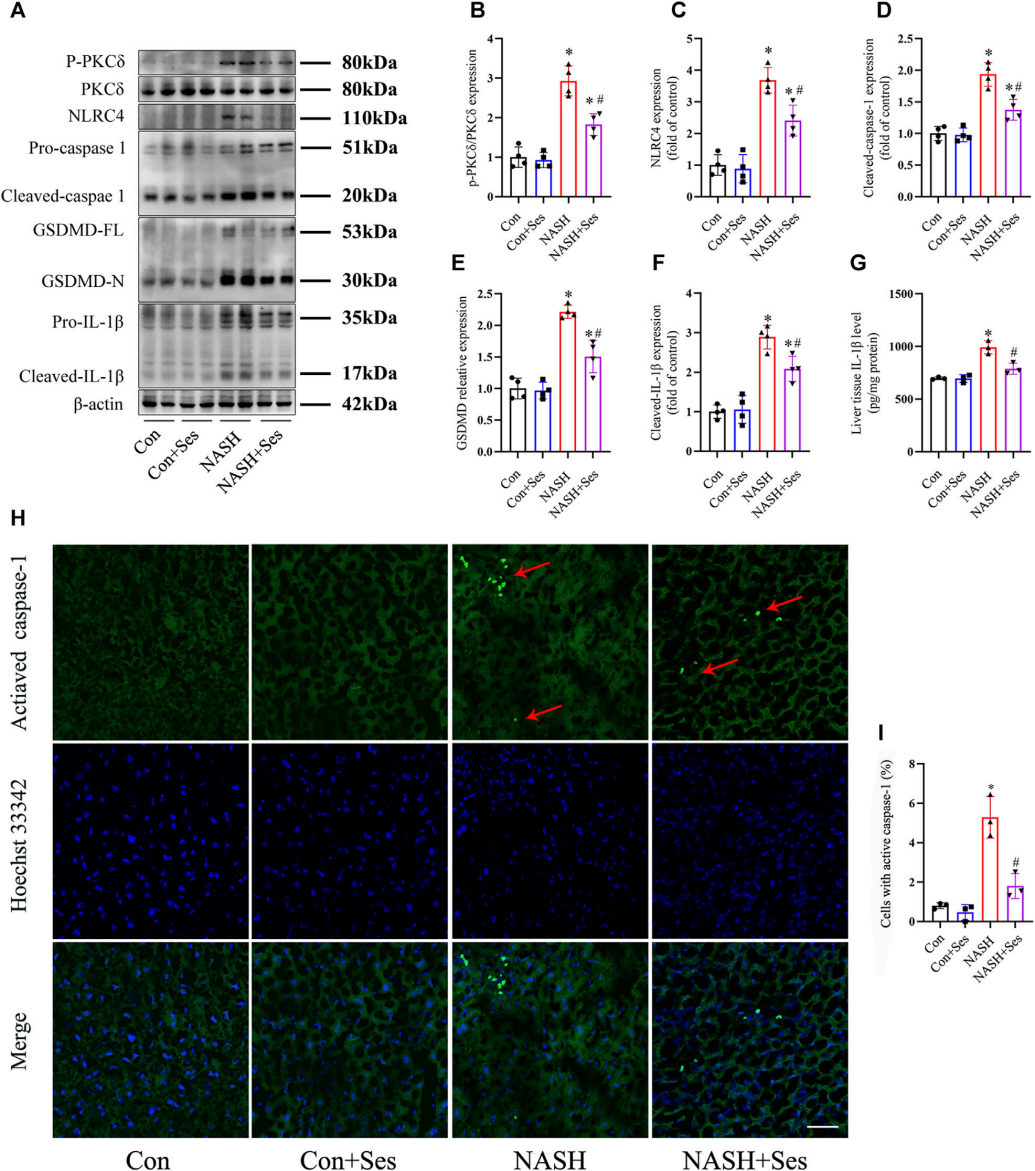
Effects of Ses on hepatic PKCδ-induced NLRC4 inflammasome activation and pyroptosis in NASH mice. **(A–F)** The panels and histograms represent the protein expression of P-PKCδ (Thr505), PKCδ, NLRC4, cleaved-caspase 1, GSDMD-N and cleaved-IL-1β in the liver tissue (*n* = 4 per group). **(G)** The IL-1β level in the liver tissue was measured using ELISA (*n* = 3 per group). **(H,I)** The activated caspase-1 (red arrow) level in the liver tissue was detected by fluorescence staining. Scale bar = 50 μm (*n* = 3 per group). *p < 0.05 vs. Con group, ^#^ *p* < 0.05 vs. NASH group.

Activation of the NLRC4 inflammasome by PKCδ induces pyroptosis in macrophages and hepatocytes (Qu et al., 2012; Koh et al., 2021). As depicted in Figures 3A, B, the ratio of P-PKCδ to PKCδ was significantly higher in the liver tissue of NASH mice than in that of control mice. Treatment with Ses significantly reduced the hepatic ratio of P-PKCδ to PKCδ in NASH mice. Furthermore, immunoblotting data revealed increased protein levels of pyroptosis markers, including NLRC4, cleaved-caspase 1, GSDMD-N and cleaved-IL-1β, in the liver tissue of NASH mice than in that of control mice. As expected, Ses treatment substantially downregulated the expression of these proteins in NASH mice (Figures 3A, C–F). Consistent with these results, Ses treatment significantly decreased the hepatic IL-1β level (assessed by ELISA, Figure 3G) and activated caspase-1 level (assessed using fluorescence staining, Figure 3H) in NASH mice.

### Effects of Ses on LDH release and mortality in PA-treated mouse primary hepatocytes

**FIGURE 4:**
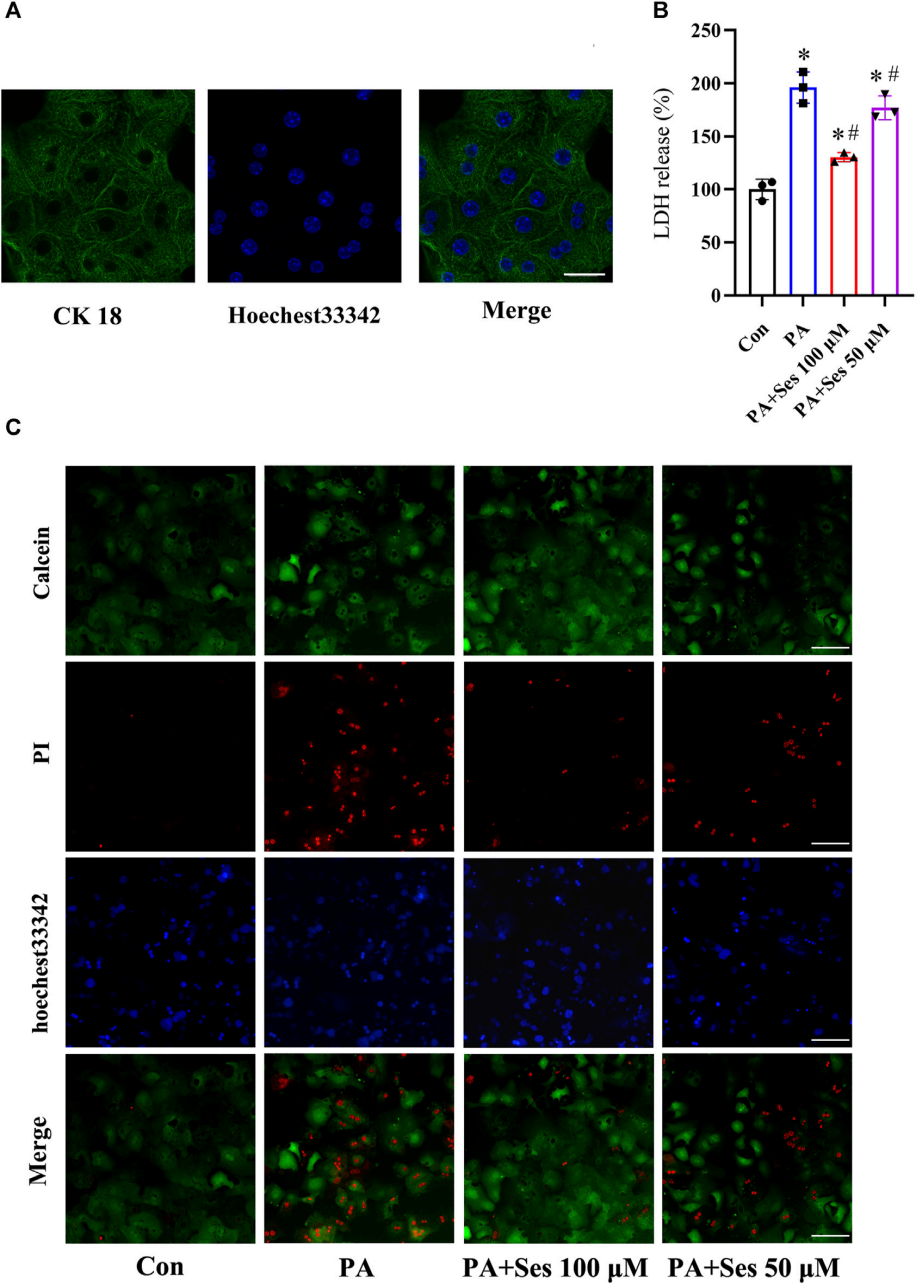
Effects of Ses on LDH release and mortality in PA-treated mouse primary hepatocytes. **(A)** Representative immunofluorescence staining photomicrographs of the liver-specific protein CK 18 in primary hepatocytes. Scale bar = 20 μm. Primary hepatocytes were stimulated with PA (0.5 mM) for 48 h in the absence or presence of 100 or 50 μM Ses. **(B)** The relative LDH release level was measured. **(C)** Calcein-AM/PI staining was employed to detect live (green fluorescence) and dead hepatocytes (red fluorescence). Scale bar = 50 μm. Data are presented as mean ± standard deviation of three independent experiments. *p < 0.05 vs. Con group, ^#^ *p* < 0.05 vs. PA group.

Immunofluorescence staining of the liver-specific CK 18 protein was utilized to identify mouse primary hepatocytes (Figure 4A). LDH leakage was monitored as a reliable indicator to assess the occurrence of pyroptosis (Shen et al., 2023; Yu et al., 2023). As illustrated in Figure 4B, the release of LDH was significantly elevated in primary hepatocytes treated with PA. Ses treatment significantly reduced the LDH levels. Additionally, Figure 4C shows that incubation with PA markedly increased the mortality rate of primary hepatocytes, and Ses treatment decreased PA-induced cell death.

### Effects of Ses on PKCδ-induced NLRC4 inflammasome activation and pyroptosis in PA-treated mouse primary hepatocytes

**FIGURE 5:**
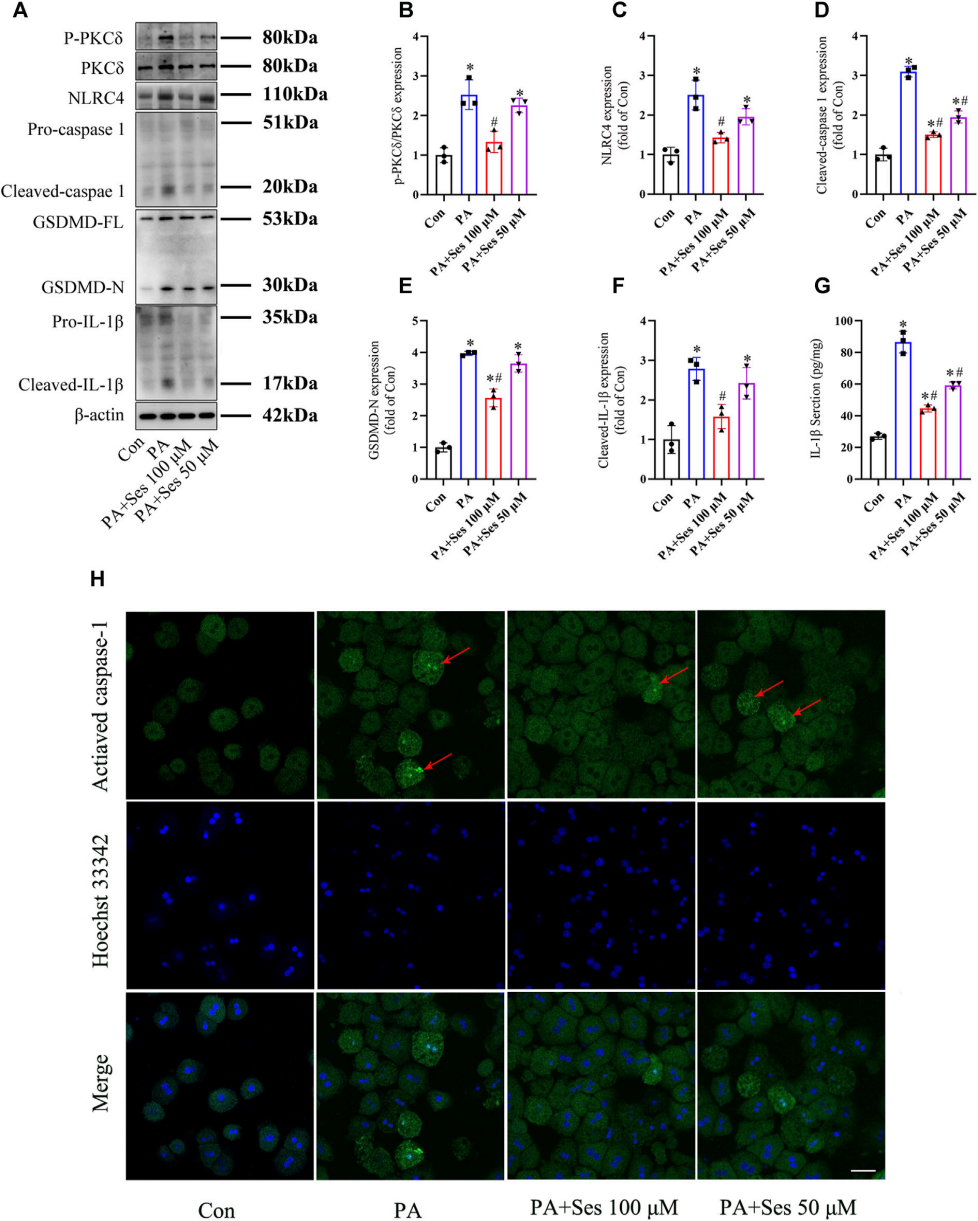
Effects of Ses on PKCδ-induced NLRC4 inflammasome activation and pyroptosis in PA-treated mouse primary hepatocytes. Primary hepatocytes were stimulated with PA (0.5 mM) and Ses (100 or 50 μM) for 48 h **(A–F)** The panels and histograms represent the protein expression of P-PKCδ (Thr505), PKCδ, NLRC4, cleaved-caspase 1, GSDMD-N and cleaved-IL-1β. **(G)** The IL-1β level was measured using ELISA. **(H)** Activated caspase-1 (red arrow) was detected by fluorescence staining. Scale bar = 50 μm. Data are presented as mean ± standard deviation of three independent experiments. *p < 0.05 vs. Con group, ^#^ *p* < 0.05 vs. PA group.

As shown in Figures 5A, B, the ratio of P-PKCδ to PKCδ was significantly increased in PA-treated mouse primary hepatocytes. Ses treatment effectively reduced the ratio of P-PKCδ to PKCδ in PA-treated primary hepatocytes. Moreover, the immunoblotting results revealed a notable upregulation in the protein levels of pyroptosis markers, such as NLRC4, cleaved-caspase 1, GSDMD-N, and cleaved-IL-1β, in PA-treated primary hepatocytes. Ses treatment significantly mitigated the expression of these proteins (Figures 5A, C–F). Consistent with these findings, Ses treatment resulted in a significant reduction in the secretion of IL-1β (as determined by ELISA, Figure 5G) and the level of activated caspase-1 (as assessed by fluorescence staining, Figure 5H) in the PA-treated primary hepatocytes.

### The anti-pyroptosis effects of Ses in PA-treated mouse primary hepatocytes are inhibited by PMA treatment

**FIGURE 6:**
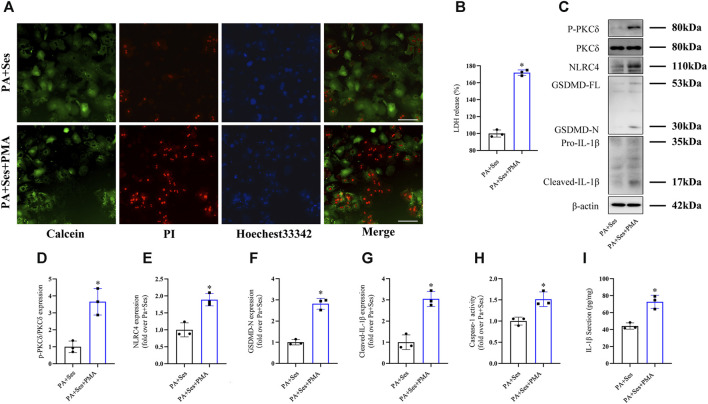
The anti-pyroptosis effects of Ses in PA-treated mouse primary hepatocytes are inhibited by PMA treatment. Mouse primary hepatocytes were stimulated with PA (0.5 mM) and Ses (100 μM) for 48 h and further stimulated with PMA (200 nM) for 12 h. **(A)** Calcein-AM/PI staining was employed to detect live (green fluorescence) and dead hepatocytes (red fluorescence). Scale bar = 50 μm. **(B)** The relative LDH release level was measured. **(C–F)** The panels and histograms represent the protein expression of P-PKCδ (Thr505), PKCδ, NLRC4, GSDMD-N and cleaved-IL-1β. **(G)** Caspase-1 activity was measured using colorimetry. **(H)** The IL-1β level was measured using ELISA. Data are presented as mean ± standard deviation of three independent experiments. *p < 0.05 vs. PA + Ses group.

To investigate the potential involvement of PKCδ in mediating the anti-pyroptosis effects of Ses, we employed the PKCδ agonist PMA (Wen et al., 2018; Jin et al., 2022). As depicted in Figures 6A, B, PMA treatment significantly reversed the Ses-induced decreases in the mortality rate and LDH release observed in the PA-treated mouse primary hepatocytes. Furthermore, PMA treatment attenuated the effects of Ses on the regulation of P-PKCδ, NLRC4, GSDMD-N and cleaved-IL-1β protein expression in the PA-treated primary hepatocytes (Figures 6C–G). Similarly, PMA treatment inhibited the Ses-induced reduction of caspase-1 activity (as determined by colorimetry, Figure 6G) and IL-1β secretion (Figure 6H) in PA-treated primary hepatocytes.

## Discussion

Due to an improved quality of life and a significant decrease in physical activity levels among individuals, the incidence of NAFLD and NASH has increased (Younossi et al., 2019). Ses is a natural lignan found in sesame oil and seeds. Ses has been shown to possess liver-protective properties, effectively improving hepatic damage in mice with high-fat and high-fructose diet-induced NASH and rats with high-fat diet-induced NAFLD (Zhang et al., 2016; Yang et al., 2023).

In our study, Ses treatment resulted in a decrease in the ratio of liver weight to body weight, as well as improvements in glucose and insulin tolerance in NASH mice with HFHCD. Furthermore, Ses administration led to reductions in the levels of ALT, AST, FFA, TC and TG. Additionally, Ses improved hepatic pathological damage and reduced macrophage infiltration in NASH mice. These findings provide further evidence that Ses effectively alleviates structural and functional liver damage in NASH**.**

Interestingly, both our present study and others suggest that Ses reduces elevated liver enzymes in NAFLD/NASH, indicating its potential role in improving liver cell damage (Zhang et al., 2016; Yang et al., 2023). However, the liver-protective effects of Ses are related to its ability to inhibit lipid metabolism and the overproduction of reactive oxygen species (Zhang et al., 2016; Yang et al., 2023). The direct effect of Ses on improving NASH-induced liver cell damage has not been fully elucidated.

Pyroptosis occurs rapidly and, as the cell volume expands, the leakage of cellular contents can lead to a severe inflammatory response (Liu et al., 2016). Consistent with findings from previous studies (Wree et al., 2014; Qiu et al., 2018; Koh et al., 2021; Meng et al., 2022), we observed significant pyroptosis, characterised by upregulated protein expression of cleaved-caspase 1 and GSDMD-N, enhanced caspase-1 activity and increased LDH release, in the liver tissue of NASH mice and in mouse primary hepatocytes treated with PA. These results suggest that hepatic cell pyroptosis plays a crucial role in the progression of NASH.

Inflammasomes facilitate caspase-1 activation, leading to the maturation and release of IL-1β, ultimately inducing pyroptosis. Ses has been demonstrated to reduce IL-1β levels both *in vivo* and *in vitro* (Phitak et al., 2012; Chiang et al., 2014; Fanhchaksai et al., 2016). Therefore, we hypothesised that Ses inhibits IL-1β production and pyroptosis by suppressing inflammasome activation.

Inflammasomes are considered multiprotein complexes, functioning as molecular switches in pyroptosis (Liu et al., 2016). NLRs form a specialised group of inflammasomes, including NLRP1, NLRP3, NLRP6 and NLRC4 (Liu et al., 2016). Preliminary experiments in this study revealed that Ses treatment led to a decrease in hepatic mRNA expression of NLRC4 in NASH mice, whereas no significant changes were observed in the mRNA expression levels of NLRP1, NLRP3 and NLRP6 (data not shown).

Ses treatment significantly decreased LDH release in PA-treated mouse primary hepatocytes. Furthermore, Ses treatment downregulated the protein expression of NLRC4, cleaved-caspase 1 and cleaved-IL-1β, effectively inhibiting inflammasome formation in the liver tissue of NASH mice and the PA-treated primary hepatocytes. Ses treatment also blocked the activation of GSDMD-N and prevented IL-1β release, thereby reducing hepatic pyroptosis both *in vivo* and *in vitro*. Collectively, these findings indicate that Met’s protective effects on liver damage may be partially attributed to its ability to alleviate pyroptosis.

In addition, Ses suppresses mast cell activation and inflammatory mediator release by inhibiting the PKCα/NF-κB signalling pathway (Zhao et al., 2018). As a member of the PKC family, increased PKCδ activation has been observed in the liver tissue of NASH mice and the human hepatic cell line L02 treated with PA (Lai et al., 2017; Koh et al., 2021). The activation of the NLRC4 inflammasome by PKCδ induces pyroptosis in macrophages and hepatocytes (Qu et al., 2012; Koh et al., 2021). Consequently, our next focus is to elucidate whether Ses’s inhibitory effects on NLRC4 inflammasome activation and pyroptosis are mediated by PKCδ.

Treatment with Ses significantly reduced PKCδ phosphorylation in the liver tissue of NASH mice and the PA-treated mouse primary hepatocytes. We subsequently employed the PKCδ agonist PMA and found that enhanced PKCδ phosphorylation by PMA significantly counteracted the Ses-induced decreases in the mortality rates and LDH release in the PA-treated primary hepatocytes. This counteraction was accompanied by an increase in NLRC4, GSDMD-N and cleaved-IL-1β protein expression as well as an elevation in caspase-1 activity and IL-1β secretion. These results demonstrate that the activation of PKCδ with PMA diminishes the protective effects of Ses against pyroptosis in hepatocytes.

The present study has certain limitations. First, Ses has been shown to suppress hepatic lipid accumulation by regulating lipogenesis and lipolysis (Pham, et al., 2023). Previous studies have indicated that lipid accumulation activates hepatic PKCδ (Lai et al., 2017; Koh et al., 2021). Therefore, further research is needed to clarify whether Ses’s anti-pyroptosis effects are associated with the inhibition of the hepatocyte PKCδ/NLRC4 axis caused by its lipid-lowering effects. Second, promoting autophagy may improve NASH by inhibiting pyroptosis (Zhu et al., 2022). Further studies are required to investigate whether Ses’s anti-pyroptosis effects are associated with its ability to induce autophagy (Tanabe et al., 2011). Finally, although Ses has been administered to patients with type 2 diabetes mellitus and hypertension (Miyawaki et al., 2009; Mohammad Shahi et al., 2017), its efficacy in treating patients with NASH remains uncertain and requires further clinical studies.

## Conclusion

**FIGURE 7:**
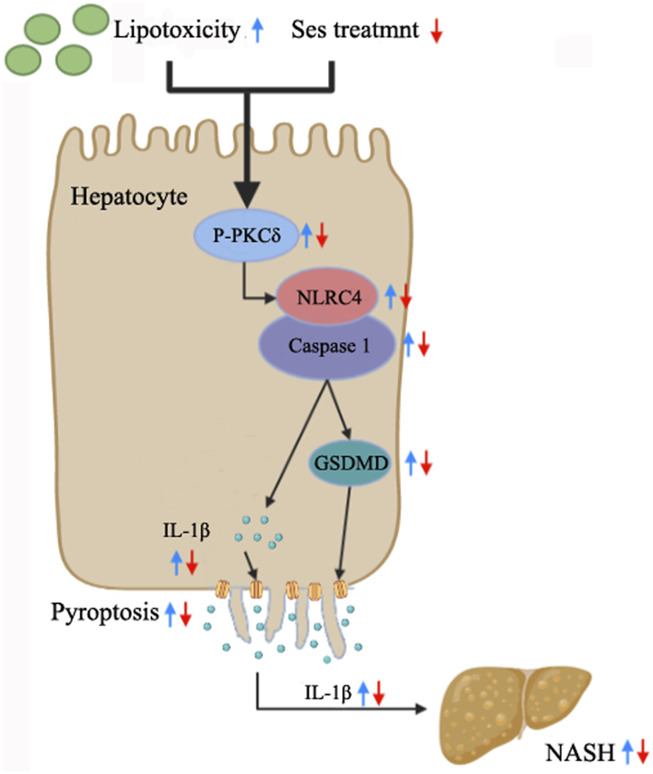
Summary of the mechanisms through which Ses ameliorates NASH by inhibiting hepatocyte pyroptosis.

The current study highlights the occurrence of pyroptosis in both NASH mice and mouse primary hepatocytes treated with PA. Additionally, it establishes, for the first time, a novel connection between the hepatoprotective effects of Ses and the inhibition of hepatocyte pyroptosis in NASH. The potential mechanisms underlying the therapeutic effects of Ses involve the modulation of the PKCδ/NLRC4/caspase-1 axis, as illustrated in Figure 7. These findings offer valuable insights into the potential therapeutic applications of Ses in NASH treatment.

## Data Availability

The original contributions presented in the study are included in the article/supplementary material, further inquiries can be directed to the corresponding authors.
